# Chitosan Hydrogels for Water Purification Applications

**DOI:** 10.3390/gels9080664

**Published:** 2023-08-17

**Authors:** Mariana Chelu, Adina Magdalena Musuc, Monica Popa, Jose M. Calderon Moreno

**Affiliations:** “Ilie Murgulescu” Institute of Physical Chemistry, 202 Spl. Independentei, 060021 Bucharest, Romania; mchelu@icf.ro (M.C.); pmonica@icf.ro (M.P.)

**Keywords:** bio-polymers, chitosan, hydrogels, adsorption, heavy metals, dyes, water treatment, wastewaters, pharmaceuticals contaminants, bio-chitosan

## Abstract

Chitosan-based hydrogels have gained significant attention for their potential applications in water treatment and purification due to their remarkable properties such as bioavailability, biocompatibility, biodegradability, environmental friendliness, high pollutants adsorption capacity, and water adsorption capacity. This article comprehensively reviews recent advances in chitosan-based hydrogel materials for water purification applications. The synthesis methods, structural properties, and water purification performance of chitosan-based hydrogels are critically analyzed. The incorporation of various nanomaterials into chitosan-based hydrogels, such as nanoparticles, graphene, and metal-organic frameworks, has been explored to enhance their performance. The mechanisms of water purification, including adsorption, filtration, and antimicrobial activity, are also discussed in detail. The potential of chitosan-based hydrogels for the removal of pollutants, such as heavy metals, organic contaminants, and microorganisms, from water sources is highlighted. Moreover, the challenges and future perspectives of chitosan-based hydrogels in water treatment and water purification applications are also illustrated. Overall, this article provides valuable insights into the current state of the art regarding chitosan-based hydrogels for water purification applications and highlights their potential for addressing global water pollution challenges.

## 1. Introduction

**Figure 1:**
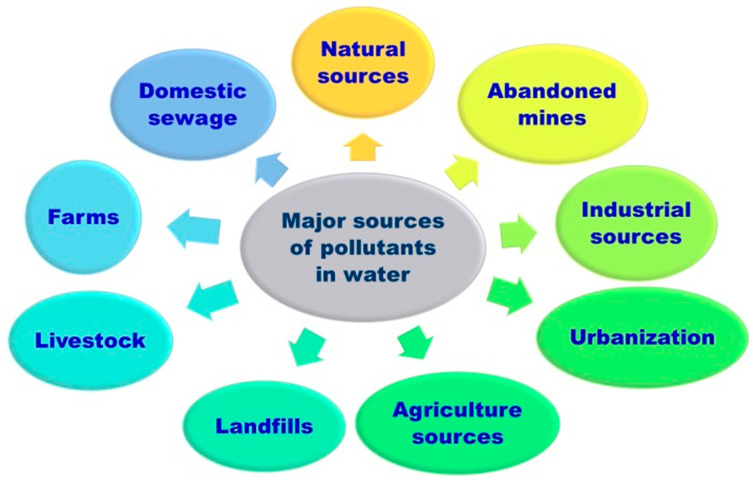
Major sources and common pathways of pollutants in water.

Life is conditioned by water for which there are no substitutes. Freshwater is a renewable natural resource, vital to the functioning of the Earth system, but present in finite quantities. Humans, plants, and animals need water to survive. Freshwater is used in agriculture, industry, power generation, sanitation, construction, transportation, and virtually in every aspect of human life. There is an imperative demand to protect freshwater ecosystems as much as possible. In addition to adapting human activities to the limits of nature’s capacity, innovative techniques are needed to protect limited water resources from pollution and to ensure the preservation of an adequate supply of good quality freshwater. With the growth of the population (which has reached the threshold of 8 billion people), the evolution of modernization, and the growth of industrialization, water pollution has become an increasingly serious problem worldwide. Various harmful pollutants such as dyes, drugs, heavy metal ions, detergents, and personal care products, derivatives or by-products of various industries (e.g., oil, mining, printers, energy, etc.) are released into the environment. Figure 1 shows the most common pathways of water pollution.

If all these pollutants are discharged into aquatic ecosystems without proper treatment, they will cause water pollution [1,2,3]. A United Nations report shows that worldwide, 2 million tons of sewage and industrial and agricultural waste are discharged every day into the world’s waterways and that 80% of the wastewater generated in developing countries is discharged without treatment into bodies from surface water [4]. A recent report also sounds a loud alarm, stating that water scarcity is becoming endemic as a result of the local impact of physical water stress, along with the acceleration and spread of freshwater pollution [5]. The first consequence of the shortage is the increased use and depletion of groundwater. It is also estimated that the groundwater depletion rate is between 100 and 200 km^3^/year, representing 15 to 25% of the total groundwater withdrawals [6]. Following the UN conference, the *United Nations Water Development Report 2023* highlights how building partnerships and strengthening action across all dimensions of sustainable development are essential to accelerate progress towards the Sustainable Development Goal on Clean Water and Sanitation (SDG 6) and to realize the human rights for drinking water and sanitation [7]. It was also pointed out that a growing number of chemicals threaten the aquatic environment. Once present in water, some of them manage to remain unchanged after treatment and persist in contaminating drinking water sources. It is estimated that in 2023, globally, there are more than 204 million substances registered in the Chemical Abstracts Service (CAS) Registry [8], of which over 350,000 chemicals and chemical mixtures have been authorized for production and use [9]. Therefore, chemicals released into the environment could cause great damage to the ecosystems, and when they reach drinking water, they become a danger to human health. It is therefore extremely imperative to adopt solutions for detecting contaminants in water, estimating risks to public health and the environment, and finding water treatment technologies.

Several technologies have been developed for water treatment, including wastewater purification and recycling, including precipitation [10,11], oxidation [12], membrane filtration [13,14,15], adsorption [16,17,18,19,20,21,22], biological treatments [23,24,25], advanced oxidation processes [26,27,28,29,30,31,32], etc. However, some of these technologies are expensive, complex, have high energy consumption, or even low efficiency. Among them, adsorption treatment is an approach that attracts the attention of scientific research for water remediation due to several attractive properties such as simple operation, low cost, high adsorption capacity, a wide spectrum of pollutants, and favorable effects. Adsorbates such as alumina [33], clays [34,35], activated carbon [36,37,38,39,40], or biomass conversion [41] are currently used in adsorption treatment. In addition, by applying appropriate adsorption–desorption methods as well as suitable adsorbents, some pollutants, such as heavy metal ions, or critical materials (e.g., rare earth elements and lithium) can be recovered [42].

In the last decade, various materials (e.g., mesoporous activated carbon, carbon-based materials, and metal-organic frameworks (MOFs)) and agricultural waste have been prepared and used for wastewater treatment and purification [43,44,45,46,47,48]. Nevertheless, their usage in these kinds of applications is still restricted due to their low performance and high cost of preparation. Natural polysaccharides present, in their structure, higher concentrations of functional groups such as –OH, –COOH, and –NH_3_ with the ability to attach or fix diverse organic or/and inorganic contaminants [49,50,51]. Recently, natural polysaccharides-based hydrogels, due to their several peculiarities of being abundant in nature, biodegradable, and biocompatible, were used as adsorbent materials for water treatment [52,53,54,55,56,57,58,59] and as flocculants for their application in drinking water purification [60,61,62,63].

Due to their excellent adsorption properties, reversible swelling, biocompatibility, biodegradability, ease of synthesis and use, as well as low price, the application of hydrogels is increasingly being considered as an alternative method in water purification. Hydrogels are hydrophilic three-dimensional (3D) crosslinked polymer networks that can adsorb and retain large amounts of water without dissolving [64,65]. Consequently, these polymer matrices adsorb water and pollutants through diffusion mechanisms and macromolecular relaxation during swelling processes [66,67].

**Figure 2:**
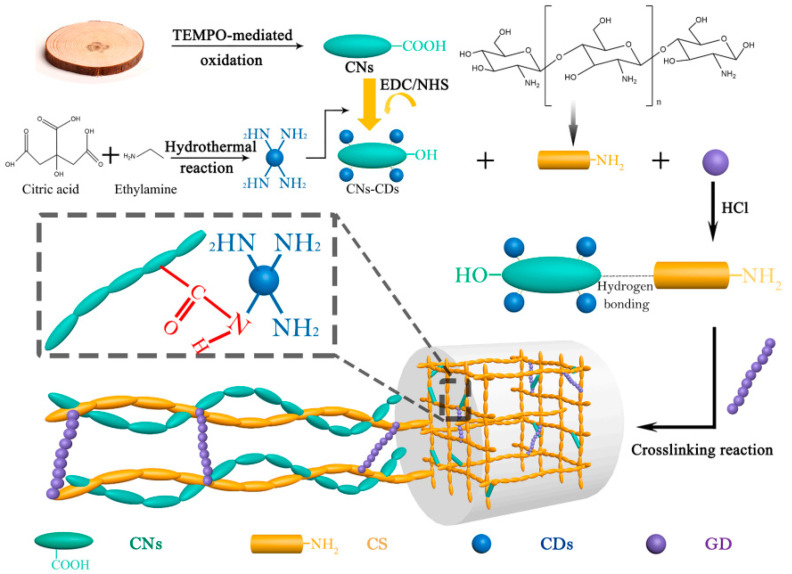
Diagram of the chitosan-based hydrogel preparation method. (Notation: CNs—cellulose nanomaterials; CS—chitosan; CDs—carbon dots; CD—glutaric dialdehyde; EDC—N-(3-dimethylaminopropyl)-N’-ethylcarbodiimide hydrochloride; NHS—N-hydroxysuccinimide). Reprinted with permission from ref. [72] Copyright 2023, Elsevier.

Distinctly, chitosan-based hydrogels are promising matrices for the treatment of polluted waters due to characteristics such as good mechanical and thermal resistance, high chemical stability, and low price and due to the accessibility of recovery—reuse of both hydrogel and pollutants [68,69,70,71]. Also, the mechanical strength of chitosan-based hydrogels can be improved by adding nanoparticles or by crosslinking with synthetic polymers or biopolymers. Due to the presence of large numbers of hydroxyl and amino groups, chitosan can easily adsorb different pollutants (e.g., heavy metals, and dyes) through hydrogen bonding and electrostatic interactions. By combining the hydrogels and chitosan advantages, it can enhance the adsorption capacity and efficiency of chitosan-based hydrogels. A schematic representation of the chitosan-based hydrogel preparation is reproduced in Figure 2 [72].

Presently, the application of polysaccharides in drinking water purification and wastewater treatment has been extensively studied [73,74], but there is still a deficiency of methodically related reviews on chitosan-based hydrogels for wastewater treatment, particularly in their adsorption of contaminants and oil–water separation. The aim of this review is to offer a comprehensive and comparative analysis of recent advancements, from the last decade, of chitosan-based hydrogels used for the adsorption of contaminants from water, providing some practical designs for researchers in solving the problem of water pollution. Also, the purpose of this review is to explore the newly developed chitosan-based hydrogels for their large-scale applications in wastewater treatment and the major features of these types of materials. From the perspective of the global goal of achieving access to quality drinking water for all, the use of chitosan-based hydrogels is an environmentally friendly, modern, and state-of-the-art method of adsorbing hazardous contaminants from wastewater.

**Figure 3:**
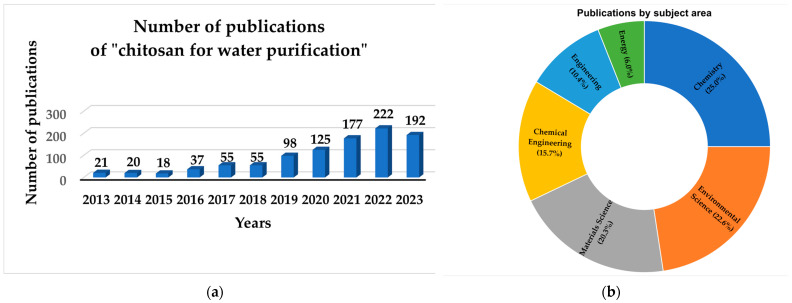

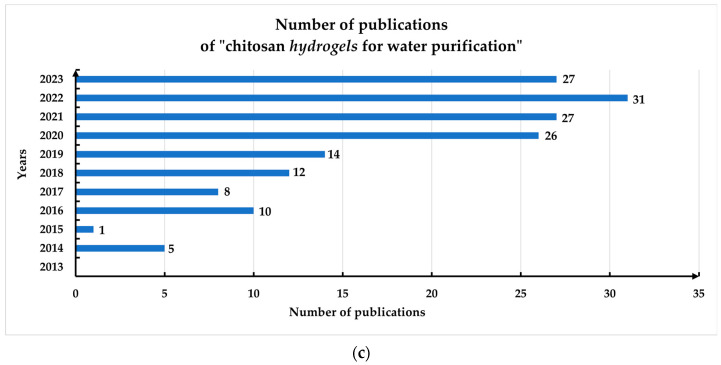
(**a**) Number of publications (articles and reviews) of chitosan for water purification from 2013 to August 2023; (**b**) publications by subject area (articles and reviews) of chitosan for water purification from 2013 to August 2023; (**c**) number of publications (articles and reviews) of chitosan *hydrogels* for water purification from 2013 to August 2023. Data were collected using the Scopus database [75].

In this regard, a review search was carried out using the Scopus database [75] in order to view the total number of publications for a period of ten years (between 2013 and the present) for bibliometric analysis. The methodological pathway was made according to the following steps: (i) the data collection step and (ii) the data visualization and graphical evaluation steps. Thus, specified keywords (e.g., chitosan, hydrogel, water, and purification) were used. The search was supplementary filtered to include only reviews and articles (in Scopus) with a publication date after 2013. Initially, the terms in all fields, article titles, abstracts, and keywords, were combined (e.g., chitosan, water, and purification) as search words. During this search, 1020 documents (articles and reviews) were found for chitosan for water purification (Figure 3a) in various areas (Figure 3b). In order to refine the search, some areas such as biochemistry, genetics and molecular biology, physics and astronomy, medicine, economics, econometrics and finance, pharmacology, toxicology and pharmaceutics, business, management and accounting, earth and planetary sciences, computer sciences, immunology and microbiology, and social sciences were excluded. Subsequently, to achieve the purpose of this review, the following keywords were combined: chitosan, hydrogel, water, and purification. The search was also filtered to include only reviews and articles published after 2013. Succeeding this research, the record of publications according to the category of “chitosan *hydrogel* for water purification” examined in this review search are smaller (161 documents) and they are presented in Figure 3c.

## 2. Adsorption Mechanisms of Chitosan-Based Hydrogels

**Scheme 1:**
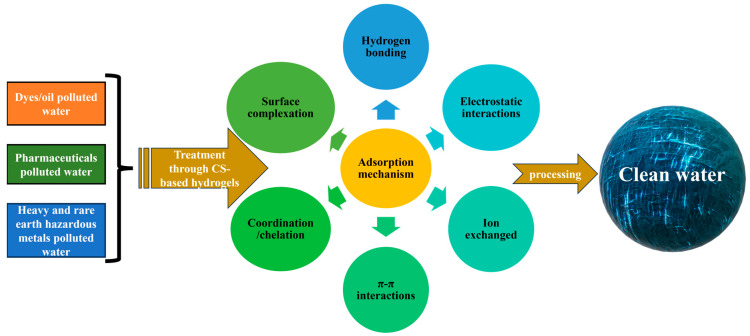
Adsorption mechanisms of pollutants from water by chitosan-based hydrogels.

The knowledge and evaluation of the adsorption mechanism of chitosan-based hydrogels is essential for further design and preparation of adsorption materials in order to increase the adsorption efficiency and capacity. Several adsorption mechanisms exist in the adsorption of pollutants from water using chitosan-based hydrogels such as hydrogen bonding, electrostatic interactions, ion exchanges, π–π interactions, coordination/chelation, and surface complexation (Scheme 1).

In Table 1, the mechanisms of adsorption, their definitions, and several examples are represented.

Due to the fact that the presence of the different functional groups on the hydrogel surface can interact physically or chemically with contaminants following one or more adsorption mechanisms, chitosan-based hydrogels are promising adsorbent materials for wastewater purification and treatment.

## 3. Categories of Chitosan-Based Hydrogels for Water Purification Applications

Chitosan (CS), a natural polycationic polymer, synthesized from partial deacetylation of chitin, is the second most plentiful natural compound. CS is a heteropolysaccharide made from D-glucosamine and N-acetyl-d-glucosamine which are linked through β-1,4 glycosidic bonds. CS has reactive chemical functional free hydroxyl and amino groups, making it a valuable bio-compound for a wide range of applications. CS is a rigid and crystalline polymer, insoluble in water at neutral pH, but due to the protonation of free amino groups, it can be dissolved in acidic conditions (e.g., acetic acid, formic acid, and citric acid). The average molecular weight of commercially produced CS is between 3800–20,000 Daltons. In addition to its antiviral and antifungal properties, the good antimicrobial activity of CS varies depending on the degree of deacetylation, molecular weight, concentration, solution pH, viscosity, and the target microorganism [82]. Another distinctive advantage of CS is its high pollutant binding capacity. It is also biocompatible and highly abundant, biodegradable and sustainable, non-toxic, and renewable and has an adaptable surface chemistry and a high surface area, and a relatively low production cost. These beneficial characteristics make CS a versatile ecological material, usable in water purification, biofiltration, pollutant removal, and wastewater treatment [83,84,85]. CS is widely used in many other applications such as in biomedicine and pharmaceutics, agriculture, environmental sensing, cosmetics, nutritional enhancement, surface biosensor applications, fabrics and textiles, veterinary medicine, and food processing. Since it is a biocompatible, fairly inexpensive material with excellent characteristics that can be used to make hydrogels with good mechanical strength, CS-based hydrogels have a huge prospect for development.

### 3.1. CS-Based Composite Hydrogels

#### 3.1.1. Composite Hydrogels Based on CS for Removing Dyes from Water

With a high degree of surface and groundwater pollution, due to dye-laden industrial wastewater, effluents pose an imminent danger to the environment and human health. A recent comprehensive review analyzed dye wastewater, stating that approximately 80% of industrial wastewater is discharged into the environment without treatment, of which 17–20% is dominated by dyes such as methylene blue (MB) and methyl orange (MO) from the textile industry. Only about 5% of textile dye is used in the dyeing process and the rest is thrown away. The study revealed that about 1.84 × 10^10^ m^3^ of dye wastewater is produced as waste from various industries such as tanneries, paper and pulp, dyes, textiles, and paints. Among these, the textile industry is considered the biggest water polluter, due to its approximately 100,000 types of dyes and a total annual production of 7 × 10^5^ tons. The same report shows an estimate for the textile industry, which would produce about 15% of the total dye wastewater or about 1000 tons of waste annually, with a concentration of 5–1500 ppm that is discharged into aquatic waters [86]. In this context, for the protection of human health and aquatic ecosystems, the treatment of discharged effluents and compliance with environmental standards require urgent measures to be taken. Since the complex aromatic structures of dyes are quite resistant to conventional biological treatments [87], various alternative methods have been developed and applied. Among them, the adsorption of dyes from wastewater is considered a promising procedure due to its operational simplicity, avoidance of additional chemical intermediates, low price, and absence of unwanted by-products [88].

From the multitude of materials proposed as adsorbents for the removal of dyes from water, composite hydrogels based on CS have been intensively studied in various applications. The main characteristics of these composites, among which are the large surface area and abundant functional groups, are favorable to the high affinity of the dye molecules to them and lead to high adsorption efficiencies [89].

**Figure 4:**
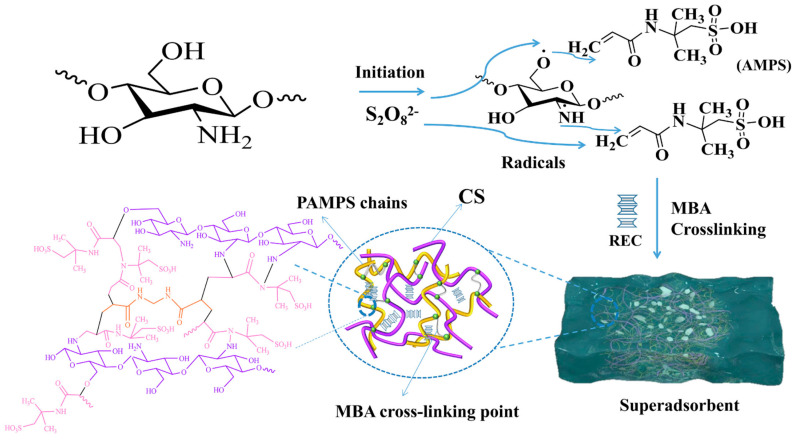
Representation of the synthesis process and the structure of the composite hydrogel. Reprinted with permission from ref. [93] Copyright 2023, Elsevier.

**Figure 5:**
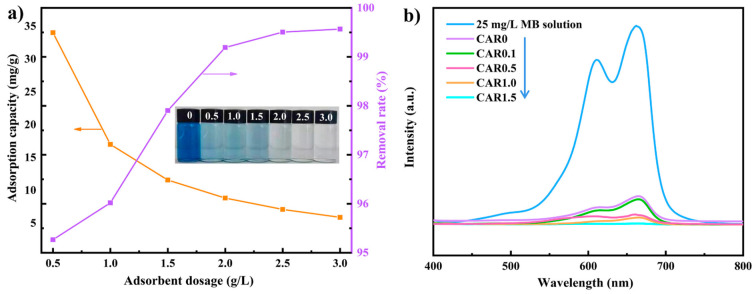
(**a**) Dependence of the adsorption capacity and adsorbent removal rate on the amount of adsorbent (initial concentration: 25 mg/L; pH 10)—inset is the digital photographs of MB solution with and without treatment with different amounts of adsorbent; (**b**) UV–Vis spectra of MB solution (25 mg/L) before and after adsorption with 3 g/L of adsorbents. Reprinted with permission from ref. [93] Copyright 2023, Elsevier.

**Figure 6:**
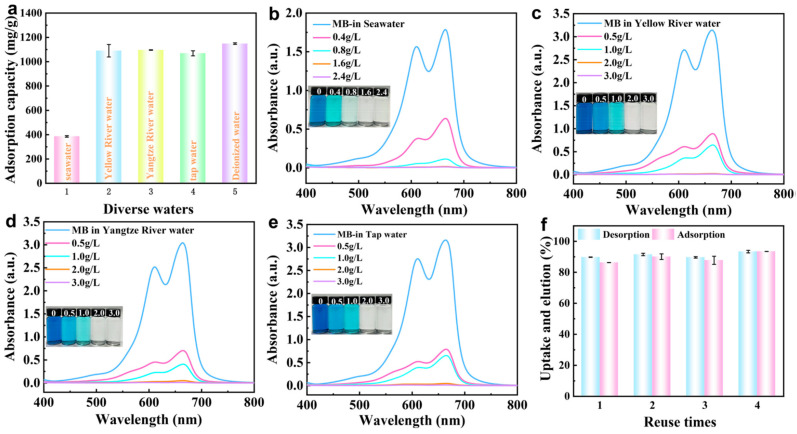
(**a**) Adsorption capacity for MB in various waters; removal rate toward MB in (**b**) seawater, (**c**) Yellow River water, (**d**) Yangtze River water, (**e**) tap water; and (**f**) adsorption capacity at different adsorption/desorption cycles (MB concentration: 500 mg/L; initial pH 10; temperature: 30 °C). Reprinted with permission from ref. [93] Copyright 2023, Elsevier.

MM Perju et al. prepared three cationic composite hydrogels based on CS and poly (N-2-aminoethyl acrylamide) (PAEA) covalently crosslinked with glutaraldehyde (GA) with different molar ratios between the primary amine groups and the crosslinker (GA/NH_2_ = 5.3 and 12.5), with the same total polycation concentration (CPC = 2 wt%) and two polycation molar ratios (PAEA:CS = 0.25 and 1.2), respectively. The synthesized hydrogels were tested as new adsorbents for two disazo dyes (Congo Red (CR) and Direct Blue 1) from aqueous solutions. Adsorption kinetic data were fitted by the pseudo-second-order equation, which gave the best correlation with the experimental data, for the studied systems. It was also observed that an increase in temperature (from 4 to 40 °C) led to an increase in the color removal efficiency for both dyes [90]. A series of carboxymethyl CS/phytic acid composite hydrogels with different amounts of phytic acid were synthesized to remove methyl orange and CR dyes from an aqueous solution. The composites exhibited high stability and were formed by co-assembly and strong crosslinking interactions of carboxymethyl CS and phytic acid. The experimental test results showed that the hydrogels have a stable porous structure with multiple wrinkles on the surface due to the formation of intermolecular hydrogen bonds between CMCS and PA during the in-situ polymerization process and that they can effectively adsorb MO and CR dyes according to the pseudo-first-order and pseudo-second-order models. By changing the different adsorption factors, namely the molar ratio between the two molecules and the pH of the solution, the optimal adsorption conditions of the composite hydrogels were obtained. Carboxymethyl CS/phytic acid composite hydrogel prepared with a 3:1 molar ratio showed the highest adsorption capacity for methyl orange (13.62 mg/g) and CR (8.49 mg/g) at pH 7 and room temperature. Also, this composite showed high adsorption reusability, stability, and fine swelling capacity [91]. A composite hydrogel was synthesized by introducing CS-crosslinked polyvinyl amine into the N, N′-methylenebisacrylamide crosslinked polyacrylic acid network for dye adsorption from an aqueous medium. The dual crosslinking network gave the hydrogel high mechanical properties, for a maximum tensile stress of up to 1.9 MPa and a strain of 92%. The adsorption capacity of MB was up to 596.14 mg/g. The hydrogel adsorption process was consistent with quasi-second-order kinetics and the Langmuir model, which demonstrated that the dye adsorption was a monolayer chemisorption process. The synthesized hydrogel presented good reusability. The adsorption efficiency for five consecutive cycles is higher than 85%. The adsorption mechanisms for the dye’s chemisorption were drawn as a monolayer, based on the well-fitted Langmuir equation, and followed a pseudo-second-order kinetic. The demonstrated good adsorption capacity is described by an adsorption mechanism based on hydrogen bonds and electrostatic interactions between the functional hydrogel’s groups and dye molecules [92]. In order to strengthen the ability of a hydrogel to remove some organic pollutants by adsorption, a method was devised to obtain it by incorporating natural rectorite into the hydrogel network based on CS-g-poly (2-acrylamido-2-methyl-propane-sulfonic acid) to form a rectorite-in-polymer network structure. Rectorite is a natural clay mineral of the interstratified natural silicate type, and it is used as an ecological adsorbent of pollutants. Figure 4 schematically represents the synthesis process and the structure of the obtained composite hydrogel. The influence of increasing the number of adsorbents on dye-removal efficiency was studied (for a MB concentration of 25 mg/L and pH 10). As the adsorbent dosage increased, the adsorption amount of MB gradually decreased, and the removal rate increased significantly (Figure 5). By introducing a lower dose of rectorite (1.2 wt%) into the hydrogel, it facilitates an improvement of its adsorption capacities towards MB in several types of aqueous media: deionized water, tap water, tap seawater, Yangtze River water, and Yellow River water (Figure 6). By incorporating a higher dose of rectorite (15.8 wt%), an improvement in the removal rate (99.6%) for MB in real waters was obtained. The adsorbent showed high adsorption efficiency in a wide pH range (2–11) and a good degree of reusability (>4 times). It was shown that the good adsorption degree of MB on the composite hydrogel was mainly determined by the driving force of the reaction (originating from the electrostatic attraction, hydrogen bonding interaction, and chemical association of the polymer network) as well as mass transfer action (ion exchange, mainly contributed by natural rectorite) [93].

A composite hydrogel as spheres prepared by encapsulating CS in an organic–inorganic iron and terephthalate ligands which develop a three-dimensional (3D) network structure was used for the adsorption of Congo red (CR) [94]. The obtained hybrid structure shows hydrolytic and good thermal stability. By varying the experimental conditions (e.g., pH, adsorbent dosage, contact time, temperature, and initial concentration), the adsorption properties were studied. The adsorption kinetics were well fitted using the pseudo-first-order model. The adsorption isotherm corresponds to the Liu model. The best adsorption performance was obtained under neutral conditions (pH 7). The adsorption capacity of Congo red has a value of 590.8 mg/g at a temperature of 298 K. The adsorption capacity of the composite hydrogel spheres was 99.97% at the adsorption equilibrium. After three adsorption cycles, the recovery was nearly 85%. The adsorption mechanism is based mainly on ionic interactions and hydrogen bonding. The authors demonstrated that the composite hydrogel spheres show valuable potential in the process of removing CR dye from aqueous media [94]. RD Hingrajiya and MP Patel incorporated magnetic Fe_3_O_4_ in CS-grafted acrylamide and N-vinylimidazole composite hydrogels by a water-mediated free radical polymerization technique with ammonium persulfate/tetramethyl ethylenediamine as the initiator as a new type of adsorbent for MB. The prepared magnetic composite hydrogel was characterized morphologically and structurally as well as by the study of the swelling behavior. Experimental adsorption tests showed that the maximum removal of MB (*q*_e_ = 860 mg/g) was obtained at pH 8, contact time 50 min, room temperature, and an initial concentration of 1000 mg/L of MB. The adsorption kinetics and adsorption isotherm models for the adsorption removal of MB on the composite hydrogel could be satisfactorily described by the second-order kinetics and the Langmuir adsorption isotherm, respectively. The composite hydrogel was proposed as a promising recyclable, durable, robust, and efficient adsorbent for the adsorption of MB from wastewater [95]. A composite hydrogel loaded with ZnO nanoparticles was prepared by grafting acrylamide over CS and gelatin polymer chains in the presence of ammonium persulfate as the initiator and maleic acid as the crosslinker. ZnO nanoparticles embedded in the CS-gelatin hydrogel were synthesized by the free radical mechanism. The nanocomposite hydrogel demonstrated a good swelling capacity, i.e., 545.81% at pH 10. The synthesized material was morpho-structurally characterized. SEM images revealed a crosslinked hydrogel network with a uniform distribution of nanoparticles, and an elemental analysis indicated the composition of elements in the hydrogel. The nanocomposite hydrogel showed good photocatalytic activity towards CR (90.8%) in sunlight and can be used as a potential photocatalyst for the photodegradation of dyes in wastewater for up to four degradation cycles. The CS-gelatin nanocomposite hydrogel showed that it can be employed as an efficient photocatalyst with good degradation capacity towards CR in wastewater [96].

**Figure 7:**
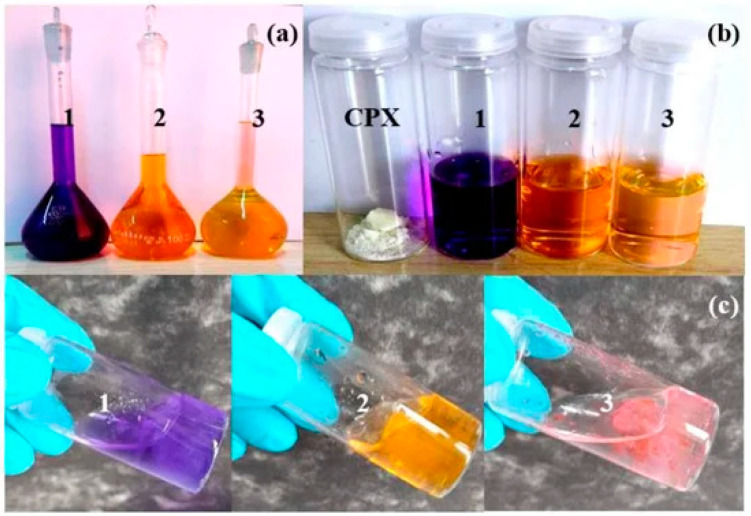
Adsorption tests of CPX hydrogel: (1) gentian violet; (2) methyl orange; (3) eosin; (**a**) stock solution; (**b**) initial time; (**c**) after 24 h [97].

Recently, we used a composite hydrogel (CPX) based on CS, polyethylene glycol, and Xanthan gum for preliminary adsorption tests of some dyes from an aqueous solution (gentian violet, methyl orange, and eosin). Since, after 24 h, the results indicated a good adsorption capacity of the synthesized hydrogel, the tests were subsequently continued with the adsorption of diclofenac sodium (DS). This experiment will be described in Section 3.1.2 (Figure 7) [97].

#### 3.1.2. CS-Based Composite Hydrogels for Pharmaceuticals Adsorption

**Figure 8:**
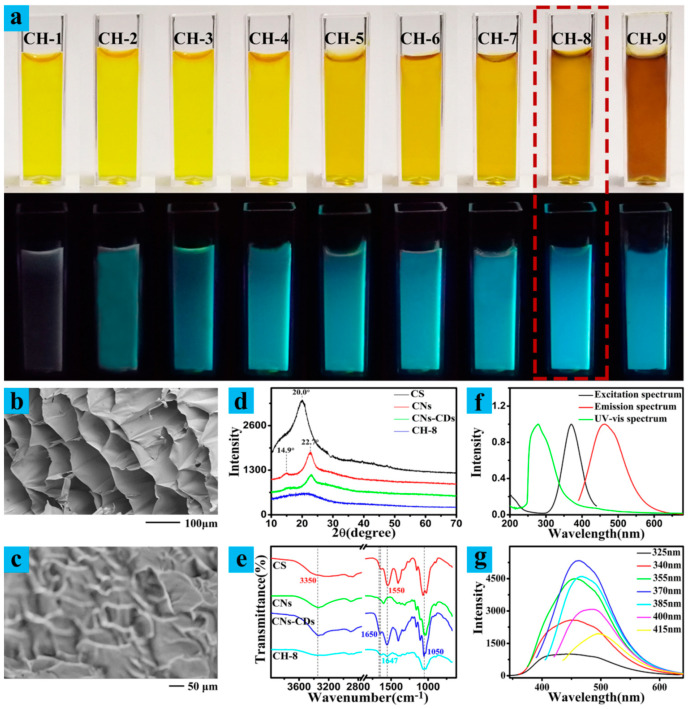
(**a**) Photo obtained under daylight and UV light of CHs with different ratios; (**b**) SEM images; (**c**) optical microscopy images; (**d**) XRD spectra; (**e**) FTIR spectra; (**f**) UV–Vis and fluorescence spectra; (**g**) excitation-dependent fluorescence behavior. Reprinted with permission from ref. [72] Copyright 2023, Elsevier.

**Figure 9:**
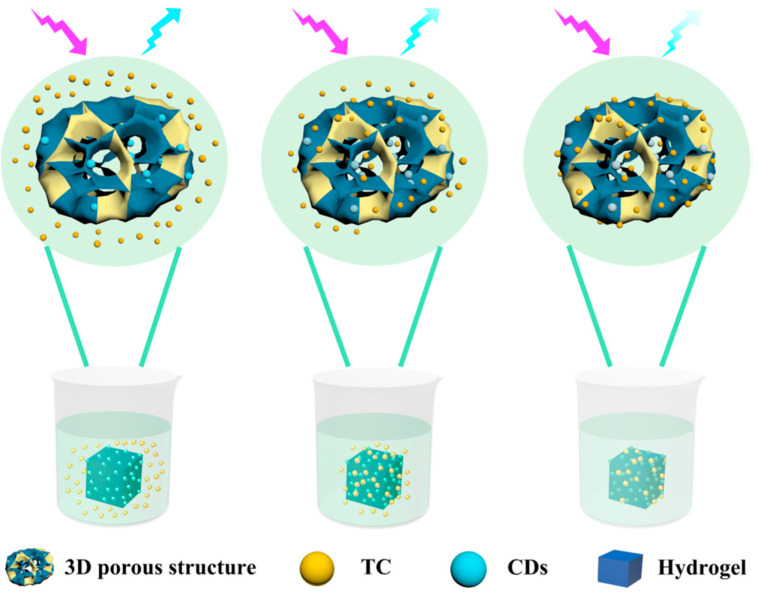
Representation of TC adsorption and detection mechanisms. Reprinted with permission from ref. [72] Copyright 2023, Elsevier.

Novel CS ionic hydrogel beads prepared by the ionic liquid impregnation of CS with tricaprylmethylammonium chloride (TCMA) were tested for the rapid adsorption of tetracycline (TC), as a model pharmaceutical compound, from an aqueous solution. Ionic liquid impregnation with TCMA greatly improved the adsorption behavior of CS toward TC, and a removal efficiency of 90% was achieved in <45 min over a wide pH range of 5–11. Increasing the amount of TCMA enhanced the adsorption efficiency of the hydrogel beads to an optimal amount (22.42 mg/g at 45 °C). The main adsorption interactions in TC adsorption on ionic beads were considered to be due to hydrogen bonds and ion-π and XH/π interactions in TC adsorption on the adsorbent surface [98]. CS/biochar hydrogel beads were explored as economical adsorbents for the removal of ciprofloxacin from aqueous solutions. Biochar was produced from the pyrolysis of yuzu pomace (pomelo peel) waste. The maximum adsorption was >76 mg/g of the adsorbent for an initial concentration of 160 mg/L ciprofloxacin. The adsorption obeyed the second-order mechanism with the main role of intra-particle diffusion and external diffusion. The adsorption capacity decreased from 34.90 mg/g to 15.77 mg/g in the presence of 0.01 N Na_3_PO_4_, unaffected by other electrolytes, such as NaCl, Na_2_SO_4_, and NaNO_3_, with the same concentration. The removal of the pollutant was achieved by a mixed mechanism, through π-π electron donor–acceptor interactions (EDAs), hydrogen bonds, and hydrophobic interactions. The adsorption decreased over time but with a slight regeneration after each adsorption cycle. The 6th adsorption was >64 ± 0.68% (25.73 mg/g), proving that the new adsorbent is an advantageous one due to its high efficiency, low cost, and ease of preparation and separation [99]. Mahmoodi et al. synthesized an interesting hydrogel adsorbent based on graphene-CS oxide and graphene-CS oxide amine, without adding chemical crosslinking agents, for the adsorption of diclofenac. Through a simple mechanical mixing method, graphene oxide-CS hydrogel (GO-CS) and amine graphene oxide-CS (AGO-CS) adsorbents were obtained. In this case, GO nanoparticles function as both the adsorbent and crosslinking agents. After a physicochemical characterization, the adsorption parameters were optimized, with the optimal mass ratio of GO to CS being 2:5, pH 5, an initial amount of 100 ppm, and a dosage of 1.5 g/L. A removal rate of approximately 90% and 97% was achieved, respectively. The results suggest that the process is of a homogeneous and monolayer type, and the data could be described by the Langmuir model [100]. HE Ali et al. prepared a nanocomposite hydrogel based on CS, polypropenoic acid, ethylenediamine (CS/PPA/EDA, with different ratios), and magnetite as a filler, using the γ-radiation technique as a crosslinking tool. The source of irradiation was gamma (^60^Co). The prepared CS/PPA/EDA/Fe_3_O_4_-NPs nanocomposite hydrogel exhibited an excellent adsorption capacity for the removal of the hazardous and toxic basic dyes Astrazon blue (AB, 193.21 mg/g) and Lerui Acid Brilliant Blue (LABB, 51.9 mg/g) from contaminated solutions. The kinetic model of the adsorption of AB and LABB dyes on the nanocomposite hydrogel obtained was verified, and it was observed that it is consistent with a second-order reaction of an endothermic nature, and the adsorption process is homogeneous and monolayer, according to the Langmuir model. The authors found that the hydrogel composite could be regenerated while retaining its properties, recommending it for wastewater remediation applications [101]. Luo et al. synthesized a new non-toxic CS-based hydrogel using cellulose nanocrystals (CN) and carbon dots (CD). The newly developed hydrogel was tested in the detection and adsorption of tetracycline (TC) from water (Figure 8). The 3D porous structure of the hydrogel was highly beneficial for TC adsorption and aggregation due to the natural CN backbone and the outstanding fluorescent feature of CDs, which were used as fluorescent probes (Figure 9). Therefore, the synthesized CH had a strong adsorption capacity and a sensitive fluorescent response. The results indicated a maximum adsorption capacity of 541.3 mg/g and a detection limit of 0.12 μg/L over the linear ranges of 0.2–1.0 μg/L and 1–200 mg/L for the selective determination and sensitivity of the antibiotic. This new hydrogel, developed as a multifunctional one, may be useful in applications for both the detection and removal of antibiotics from contaminated waters [72].

**Figure 10:**
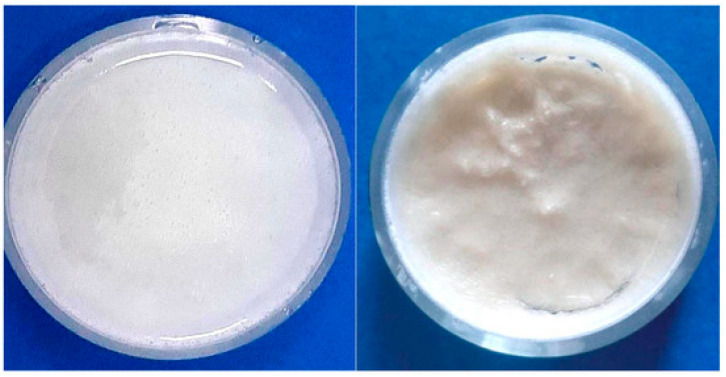
Optical images of CPX hydrogel: wet (**left**) and dry (**right**) [97].

**Figure 11:**
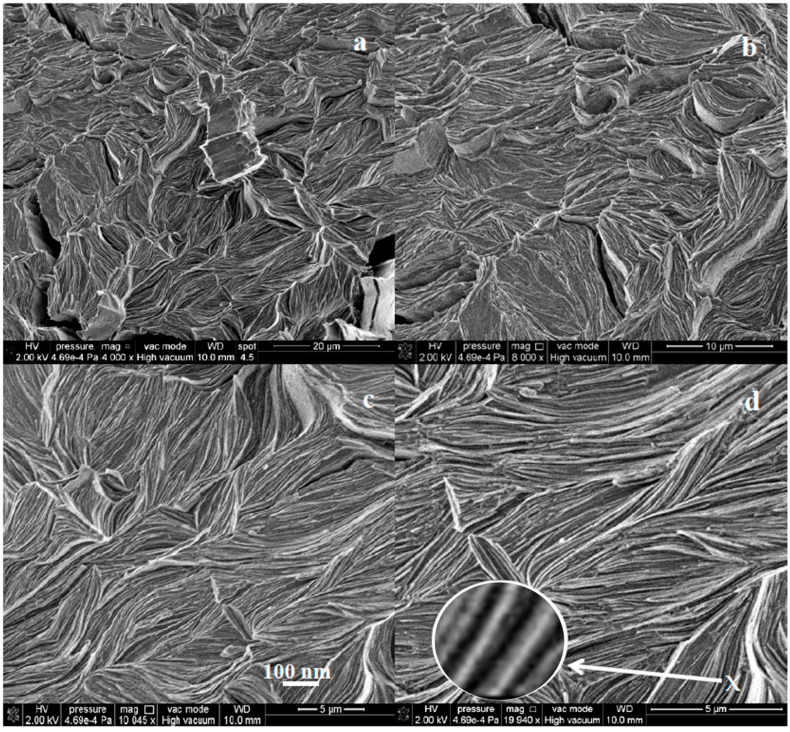
SEM micrographs at different magnifications showing the microstructure of CPX hydrogel; (**a**,**b**) bundles of 2D stacked sheet-like structures, (**c**,**d**) magnified images (below 100 nm); a magnified image (300,000×), at the position marked with an ‘x’ [97].

In our recent work, we prepared a new composite hydrogel based on CS, polyethylene glycol, and Xanthan gum, named CPX, by a green synthesis as an economical adsorbent for diclofenac sodium (DS) in wastewater (Figure 10) [97]. The SEM analysis revealed an advantageous two-dimensionally ordered structure, which can lead to the improvement of the swelling capacity of the hydrogel by about 83% after 360 min (Figure 11). The swelling analysis demonstrated that it is not dependent on pH. The experiments were made at different pH values (pH 3, pH 7, and pH 9). The results showed that the swelling degree after 4 h is 83%, independent of pH values, in the limit of experimental errors [97]. The hydrogel showed an adsorption capacity (172.41 mg/g) at the highest amount of the adsorbent (200 mg) after 350 min. The adsorption kinetics followed the pseudo-first-order model, while the adsorption mechanism was explained by the Langmuir and Freundlich models. The synthesized hydrogel could be used as a promising, eco-friendly, and cost-effective adsorbent for the removal of DS from wastewater [97].

J Tang et al. performed a very interesting study for the adsorption of norfloxacin (NOR), a broad-spectrum antibiotic that is used as a treatment for both humans and animals [102]. Once in the aquatic ecosystem, through various pathways, even in small amounts, NOR residues can cause the generation of antibiotic-resistant bacteria, endangering public health. For this reason, it is very important to remove NOR from the aquatic environment. Calcium alginate (CA)/CS hydrogel precursor spheres were prepared. Acid-washed spheres (CA/CS-M) demonstrated a much better adsorption of NOR (310.6 mg/g) than the precursor CA/CS spheres. Even though acid washing was expected to remove CS from CA/CS hydrogel spheres to obtain a higher specific surface area, as scanning electron microscopy and a BET assay showed, a small part of CS remained, increasing the structural stability of the material and leading to a more negatively charged surface characterized by Zeta potential. This feature is the main reason for the increase in the fast adsorption capacity of NOR. The maximum NOR adsorption capacity of CA/CS-M hydrogel spheres was 4.5 times higher than that of the untreated hydrogel spheres. It was demonstrated by pH and density functional theory calculations that NOR was adsorbed on the CA/CTS-M hydrogel spheres mainly through electrostatic interactions. The morphology of the adsorbent did not change significantly after 15 adsorption cycles, and the adsorption performance remained very good. The prepared hydrogel spheres have a great potential to be used as eco-friendly and extremely stable adsorbents for pollutants such as norfloxacin in the aqueous environment [102].

#### 3.1.3. CS-Based Composite Hydrogels as Biosorbent of Heavy and Rare Earth Hazardous Metals

Lead is known to be a toxic heavy metal that is very harmful to both humans and the aquatic environment. The development of fast, efficient, inexpensive, and environmentally friendly materials for the treatment of lead-contaminated wastewater is a challenge [103]. RM Vieira et al. investigated the adsorption and removal potential of Pb^2+^ and Ni^2+^ ions from aqueous solutions of two prepared hydrogels [104]. A CS-based hydrogel and a CS/acid-activated montmorillonite composite hydrogel were prepared by copolymerizing CS, acrylic acid, and N, N′-methylene bisacryl amide. The adsorption capacities of the dry CS-based hydrogel for Pb^2+^ and Ni^2+^ ranged from 41.06 to 30.20 and from 42.38 to 36.45 mg/g in the pH range between 5.5 and 3.5. The adsorption capacities of CS/acid-activated montmorillonite composite hydrogel ranged from 35.22 to 26.11 and from 37.16 to 42.04 mg/g in the pH range between 5.5 and 3.5. The adsorption mechanism of the two pollutant ions was evaluated, and the best kinetic fit for the adsorption of Pb^2+^ and Ni^2+^ ions to both hydrogels was observed using the second-order nonlinear kinetic model, indicating that the limiting phase of the adsorption process involves chemical bonding through the sharing of electrons between the adsorbent and the adsorbate [104]. Metal ion-modified hydrogels have been observed to greatly improve their pollutant removal and recovery capacity compared to that of regular hydrogels. In a recent study, H Zhao et al. synthesized a hydrogel based on sodium alginate/CS/copper ion nanofibrillar cellulose (SCC-Cu) by a semi-dissolved acidification sol-gel transition method with an internal gelation method. Also, an SCC-CuMOF composite was prepared by the in situ growth of Cu-based metal-organic frameworks (MOFs) on the surface of the SCC-Cu hydrogel [105]. The obtained materials were used to remove Pb^2+^ from aqueous solutions. The effects of pH, coexisting ions, contact time, initial ion concentration, as well as temperature on Pb^2+^ removal were investigated. The prepared composite material showed excellent pollutant ion adsorption performance according to the pseudo-second-order model and the Langmuir model, with the maximum adsorption capacity being 531.38 mg/g. The adsorption thermodynamic study suggested that the adsorption process was spontaneous and endothermic. The authors suggested that Pb^2+^ could be adsorbed on the composite material through ion exchanges and coordination interactions [105].

Among the multitude of metallic pollutants, chromium is found natively in the environment, as it is a part of the composition of natural minerals. In nature, chromium exists in two forms: trivalent and hexavalent. If Cr(III) can be assimilated as a “beneficial” ion, because it is an essential micronutrient required for the proper functioning of many biological cycles, Cr(VI) is a dangerous element extremely toxic to all biological systems, including humans and animals. Due to bioaccumulation, it can cause genetic mutations along the food chain. Therefore, the Cr content must be strictly monitored, especially in surface and groundwater and in drinking water sources. The maximum permissible limits for total Cr content recommended by the WHO are 50 µg/L, while the European Union (EU) has set a limit value of 100 µg/L for total chromium in drinking water as part of its Drinking Water Directive. For hexavalent chromium in drinking water, the indicative value established by the WHO provisionally is 0.1 µg/L, while the maximum limit for the discharge of hexavalent chromium into the aquatic environment according to the European Union is 0.5 µg/L [106]. PB Vilela developed a hydrogel synthesized by the chemical crosslinking of CS, polyacrylic acid, and N, N′-methylene bisacryl amide as a cost-effective and environmentally friendly alternative adsorbent for the treatment of water and wastewater containing heavy metals [107]. It was tested in adsorption and removal studies of chromium (VI) ions contained in aqueous solutions. The maximum chromium (VI) adsorption capacities of the dry hydrogel were 73.14 and 93.03 mg/g, according to the Langmuir and Sips nonlinear isotherm models, respectively. The best fit for the adsorption of Cr(VI) ions to the CS-based hydrogel was found with the nonlinear Redlich–Peterson isotherm model. The removal percentage of chromium (VI) at pH 4.5 and the initial metal concentration of 100 mg/L was 94.72%, the adsorption mechanism of Cr(VI) being one governed by the simultaneous formation of monolayer and multilayer interactions. The best kinetic fit for the adsorption of Cr(VI) ions to a CS-based hydrogel was found with the pseudo-nth-order non-linear kinetic model [107]. S Wankar et al. fabricated a silver-CS (Ag-CS) nanocomposite hydrogel for the rapid detection and removal of hexavalent chromium from water [108]. It was prepared at a low cost by an in situ reduction of AgNO_3_ to silver nanoparticles, which were embedded in a highly porous CS-based hydrogel matrix network obtained by crosslinking under ambient conditions. The low-cost hydrogel obtained was used for the rapid detection and removal of hexavalent chromium from water. This method was chosen to ensure a high stability of the AgNPs. The Ag-CS nanocomposite hydrogel acted as a colorimetric sensor for Cr(VI) with a detection limit as low as 0.1 ppb and demonstrated a fairly high removal efficiency of 83% for Cr(VI) within a rapid 30 min interval [108].

S-C Yang et al. developed inexpensive high-performance biosorbents from abundant and renewable natural polymers (cellulose and CS) for water purification and the removal of metal ions from water [109]. The researchers synthesized cellulose-CS composite hydrogels through a co-dissolution and regeneration process using a hydrate molten salt (a 60 wt% LiBr aqueous solution) as a solvent. The addition of CS increased the functionality for metal adsorption and the specific surface area and improved the mechanical strength of the composite hydrogel, compared to the pure cellulose hydrogel. The composite hydrogel with 37% cellulose and 63% CS demonstrated an adsorption capacity of 94.3 mg/g (1.49 mmol/g) towards Cu^2+^ at 23 °C, pH 5, and an initial metal concentration of 1500 mg/L, which was 10 times higher than the adsorption capacity of pure cellulose hydrogel. The metal adsorption capacity of the hydrogel was pH-dependent and was stable under slightly acidic conditions (pH 3–6) but significantly decreased under strongly acidic conditions (pH < 3) due to the protonation of the amino group and the dissolution of CS. From a mixture of metals in a solution, the cellulose-CS composite hydrogel showed a selective adsorption of metals in the order of Cu^2+^ > Zn^2+^ > Co^2+^ [109].

**Figure 12:**
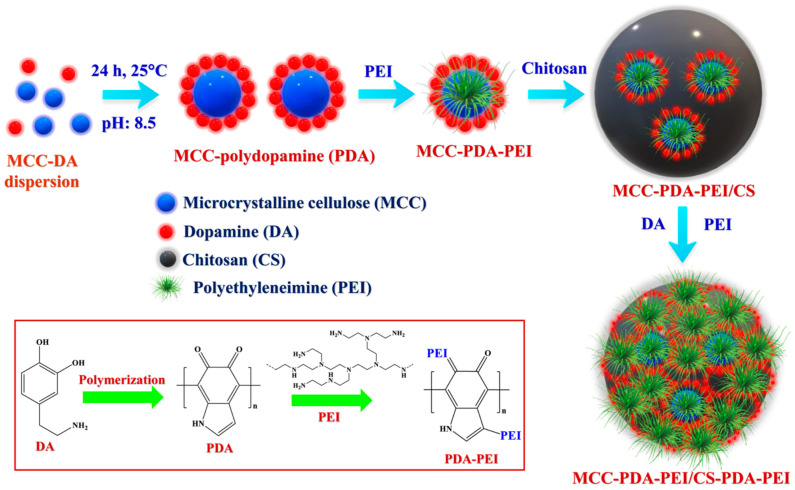
Graphic representation of MCC-PDA-PEI/CS-PDA-PEI hydrogel. Reprinted with permission from ref. [110] Copyright 2023, Elsevier.

CB Godiya et al. used CS to develop a new environmentally friendly bilayer amine group embedded microcrystalline cellulose (MCC)/CS hydrogel, synthesized by the integration of polydopamine (PDA) and polyethyleneimine (PEI) (Figure 12) [110]. The hydrogel was investigated for the reliable and efficient extraction of copper (Cu^2+^), zinc (Zn^2+^), and nickel ions (Ni^2+^) in effluents. The MCC-PDA-PEI/CS-PDA-PEI hydrogel exhibited very good Cu^2+^, Zn^2+^, and Ni^2+^ adsorbances of ~434.8, ~277.7, and ~261.8 mg/g, respectively, in a single ion adsorption due to the abundant adsorption sites. The adsorption kinetics and isotherm conformed to pseudo-second-order and Langmuir models, respectively, indicating monolayer chemisorption. In a mixture of several ions in a solution, the hydrogel removes metal cations but with a slightly higher selectivity for Cu^2+^. In several adsorption/desorption cycles, the hydrogel retained >40% metal ion adsorption and desorption capacities after four consecutive cycles. Furthermore, the in situ reduction of adsorbed Cu^2+^ formed a hydrogel patterned with Cu nanoparticles that showed excellent catalytic activity in the hydrogenation of 4-nitrophenol (4-NP) to 4-aminophenol. The newly fabricated hydrogel can be described as both a bioadsorbent and an efficient biocatalyst for the extraction of heavy metal ions and the catalytic hydrogenation of 4-NP in contaminated water [110].

C Li et al. obtained a CS-based fluorescent hydrogel through a three-step synthesis strategy using two dialdehydes [111]. NO_2_-boron-dipyrrolemethene (BODIPY) was prepared first, and then the -NO_2_ group was reduced to the -NH_2_ group. Finally, NH_2_-BODIPY was introduced into CS via the Schiff base formation reaction via bi-aldehyde. The prepared fluorescent CS hydrogels were used for the selective detection and adsorption of Hg^2+^/Hg^+^ in aqueous media. The adsorption capacity of the fluorescent hydrogel is 121 mg/g of mercury ions, almost seven times higher compared to the unmodified hydrogel, with a detection limit of 0.3 μM. The Hg^2+^/Hg^+^ elimination isotherm and kinetics follow the Langmuir isotherm and pseudo-second-order kinetics, respectively [111]. DC Mota Ferreira et al. successfully synthesized complex polyelectrolyte granular structures formed by the interaction of CS and carboxymethylcellulose (CMC), namely CS/CMC macro-PECs [112]. They were tested as adsorbents for six pollutants commonly found in wastewater: sunset yellow (SY), MB, CR, safranin (S), cadmium (Cd^2+^), and lead (Pb^2+^). The optimum adsorption pH values at 25 °C were determined to be 3.0, 11.0, 2.0, 9.0, 10.0, and 9.0 for SY, MB, CR, S, Cd^2+^, and Pb^2+^, respectively. The prepared adsorbent demonstrated an excellent adsorption capacity for the removal of SY, MB, CR, and S dyes, as well as the heavy metal cations Cd^2+^ and Pb^2+^ from aqueous media. Adsorption kinetics showed that the processes, depending on the type of adsorbate, followed pseudo-first-order or pseudo-second-order models. The maximum adsorption capacity of CS/CMC macro-PECs for the removal of SY, MB, CR, S, Cd^2+^, and Pb^2+^ was 37.81, 36.44, 70.86, 72.50, 75.43, and 74.42 mg/g, respectively (corresponding to 98.91%, 94.71%, 85.73%, 94.66%, 98.46%, and 97.14%). Adsorbent granular structures can be regenerated after adsorption of any of the six studied pollutants and can be reused [112]. A very interesting recent study presented the adsorption of europium ions from water [113]. This rare earth ion is used in the manufacturing of superconductors, fluorescent lamps, and in various commercial uses, and radioactive isotopes of europium are employed in nuclear medicine for radiation therapy and biomedical imaging [114]. It is also involved in the manufacturing of nuclear fuels. Sometimes, relatively small amounts of liquid nuclear waste can reach the environment. The application of adsorbent materials for the treatment of liquid radioactive waste is a feasible and effective procedure due to its high efficiency, low cost, selectivity, and accessibility, adsorption being considered one of the most advantageous methods of radionuclide extraction. The paper presented the creation of a hydrogel obtained by copolymerizing acrylamide and maleic acid on the surface of CS using gamma radiation. The adsorption of Eu ions from an aqueous solution was initially fast, with the hydrogel showing a maximum adsorption capacity of 144.96 mg/g. The adsorption kinetics were described by the pseudo-second-order model, and the adsorption process was spontaneous, exothermic, and favorable at a lower temperature. An efficient desorption of Eu ions was achieved in the presence of 0.1 M HCl and AlCl_3_ (97.09% and 88.63%, respectively) [113].

Table 2 summarizes the latest developments in CS-based composite adsorbent hydrogels for the removal of pollutants from wastewater.

### 3.2. CS-Based Beads Hydrogels

Various adsorption mechanisms (electrostatic interactions, complexation, hydrophobic interactions, coordination/chelation, pore filling, ion exchange interactions, and hydrogen bonding) between sorbent and sorbate are involved during the adsorption of different pollutants from wastewater by means of CS beads [135].

There are numerous CS beads used for the removal of pollutants from waters: CS-based composite beads, functionalized CS beads, metal–organic framework-based CS beads, magnetic CS beads, co-polymeric CS beads, and imprinted CS beads. A brief summary of the types of CS beads and their main advantages and disadvantages and the involving mechanisms are presented in Table 3.

Here, the preparation, adsorption mechanisms, and capacities of several CS-based beads’ hydrogels are extensively examined. Recently, it has been shown that CS has an increased capacity to cover alginate beads with an influence on the diffusion rate of the encapsulated compounds or with the proven action of being an excellent additive with the role of modifying the bead structure [160]. Bioadhesive, biodegradable, and biofunctional alginate-CS beads were developed by an ionotropic gelation preparation method using dysprosium (Dy^3+^) ion as the crosslinker for the effective removal of anionic (Fluorescein) and cationic (Rhodamine B) dyes. Adsorption kinetic studies demonstrated that the adsorption process is controlled by a chemisorption mechanism. Physical, chemical, and rheological analyses confirmed the stable and excellent mechanical properties and demonstrated the potential of the obtained hydrogel to be used for effective wastewater treatment [160]. The newly produced amidoxime-grafted sepiolite-based CS composite beads as an organic–inorganic-hybrid system was used for the removal of dyes and toxic metals from wastewater [161]. CS-amidoxime-grafted sepiolite composite beads were thermally stable, had good mechanical properties and presented an excellent adsorption capacity (99 mg/g for copper ions). The adsorption process is governed by a homogenous mechanism with the formation of a monolayer. A novel polymeric material obtained from CuO nanoparticles synthesized by a green method from *Ficus retusa* plant extract and embedded in the CS bead matrix was used for the removal of CR and eriochrome black T organic dyes with an adsorption capacity of 119.7 mg/g for CR and 235.7 mg/g for eriochrome black T [162]. The results showed a 97% removal of the dyes in a time interval of 4 h by an adsorption process at equilibrium, characterized by the Freundlich isotherm model. These CS composite beads can be reused for up to five cycles.

**Figure 13:**
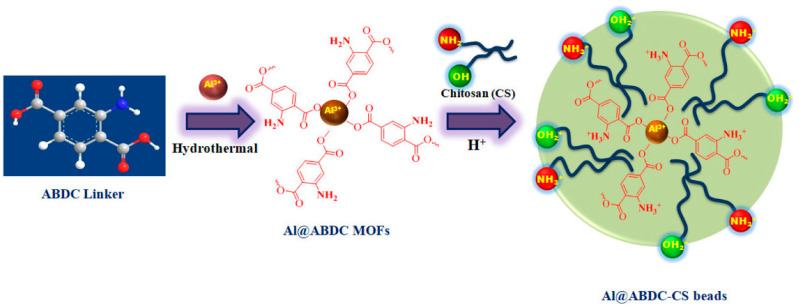
Representation of the formation mechanism of organic aluminum (Al) frameworks functionalized with 2-aminobenzene-1,4-dicarboxylic acid (ABCD) and incorporated into hybrid CS beads. Reprinted with permission from ref. [164] Copyright 2023, Elsevier.

A novel imidazolium-functionalized polysulfone/diethylenetriaminepent–acetic acid—CS bifunctional composite bead was successfully created using the phase inversion method [163]. The material was useful for the simultaneous removal of two heavy metal cations (Cr(VI) and Cu(II)) from complex wastewater. The maximum adsorption capacities obtained at pH 5 were 56.72 mg/g for Cr(VI) and 43.05 mg/g for Cu(II). The adsorbent showed good reusability in the removal process. The adsorption isotherms were based on a Sips model which is a hybrid between Freundlich and Langmuir isotherms and which demonstrated that the adsorption process of Cr(IV) and Cu(II) ions is not represented by a simple monolayer adsorption but by a heterogenous one. Removing fluoride from drinking water is a vital process. In this regard, materials with a high degree of selectivity with high porosity and an improved surface area were represented by metal organic frameworks. Organic aluminum frameworks functionalized with 2-aminobenzene-1,4-dicarboxylic acid and incorporated into CS hybrid beads were designed for water defluoridation (Figure 13) [164].

The maximum defluoridation capacity was found to be 4.9 mg/g for a contact time of 20 min and for neutral conditions. The best fit of the fluoride adsorption model was observed for the Langmuir isotherm, leading to an adsorption mechanism based on complexation and electrostatic interactions. The obtained adsorbent can be reused for up to six cycles.

Several studies have demonstrated that CS bead-based magnetic adsorbents have improved stability and the ability to prevent the oxidation process, facilitate adsorbent separation, and lead to a total adsorption of pollutants through electrostatic interactions and complexation mechanisms [165,166]. A cellulose–CS–magnetite–alginate composite hydrogel bead was developed for the treatment of heavy metal industrial effluents [167]. The synthesis of a magnetic iron oxide tethered CS-GO nanocomposite for the removal of cationic MB dye from wastewater was reported. The maximum adsorption capacity at pH 7.4 and a contact time of 300 min was 74.93 mg/g. The adsorption kinetics fit the pseudo-second-order model, and the adsorption capacity at equilibrium was well described by the Freundlich adsorption model. The reuse was evaluated up to four successive cycles at different pH values with a good adsorption capacity [168]. Using a mixture of CS and magnetite in sodium alginate solution, a magnetic hydrogel of CS beads was obtained and used for the removal of Cu(II) ions from wastewater. At a pH value of 5 and a contact time of 24 h, the adsorbent obtained showed the maximum adsorption capacity and a removal efficiency of 56.51% [169]. Reusable and stable hydrogel-based magnetic double-network CS/CA beads were prepared and used for surfactant adsorption and MB dye removal from water [170]. The adsorption capacity of the prepared hydrogel beads (surface area of 0.65 m^2^/g) was 1.9 mg/g for the removal of cationic hexadecylpyridinium chloride and 1.2 mg/g for anionic sodium dodecyl sulfate, respectively. Adsorption kinetics followed a pseudo-second-order and Freundlich isotherm models. The adsorption process showed an efficiency of 61% in removing MB dye from an aqueous solution.

Few research studies are focused on printing technology to obtain CS-based beads. The detection of target ions using printing technologies is an efficient method due to the memory effect obtained from the preparation method. Ion-imprinted montmorillonite nanosheets/CS gel beads with a porous structure were synthesized using the ion imprinting technique for the selective adsorption of Cu(II) ions [171]. The Redlich–Peterson isotherm adsorption model was found to be suitable to describe the adsorption process. A cation exchange mechanism was found for Cu(II) ion adsorption based on SEM-EDS and XPS studies. Adsorbent performance has an efficient removal and recovery of copper ions from wastewater after five cycles. The synthesis of a new type of ion-imprinted macroporous honeycomb structured as CS-based kaolin clay composite foams using both freeze-drying and ion-imprinting methods was described by Yu et al. [172]. The synthesized ion-imprinted composite foams were used for the selective biosorption of U(VI) from wastewater. FTIR and XPS structural analyses revealed an excellent honeycomb-like structure with abundant accessibility of hydroxyl and amine functional groups. Adsorption isotherm studies indicated a maximum capacity evaluated as 286.85 mg/g for U(VI) at 298 K and pH 5, evaluated for the Langmuir isotherm model and kinetics displayed by a pseudo-second-order model. The reusability of the adsorbent is five cycles. The imprinted CS beads exhibited an outstanding adsorption capacity and superior recognition ability of the target pollutants in wastewater. Nevertheless, further research is needed to understand the interactions and preparation mechanisms for the emerging effective imprinted CS beads. Also, much attention must be paid to the development of efficient and inexpensive preparation procedures to guarantee the physical, mechanical, and chemical stability of CS beads.

### 3.3. CS Graft Copolymeric-Based Hydrogels

Another advantageous alternative method to functionalize the surface of natural polymers is graft copolymerization, which can be initiated by chemical methods, radiation techniques, or by other methods, such as enzymatic grafting. A molecular design specifically tailored for the targeted adsorption application can be achieved by grafting functional polymers onto the polysaccharide backbone. This is an effective method for structural modifications of hydrogels with desired functional groups and for obtaining materials with superabsorbent potential [173]. By adding functional groups such as amino, sulfur, carboxyl, and alkyl, the adsorption capacity of hydrogels can be improved. The most important parameters influencing the grafting percentage are initiator type, initiator concentration, grafting time, monomer type, monomer concentration, and temperature [174,175,176,177,178]. Other factors that can influence the properties of grafted or functionalized CS are the molecular structure, length, and number of side chains [179]. A chemical grafting requires chemical initiators (e.g., potassium persulfate, ammonium persulfate, ceric benzoyl peroxide ammonium nitrate, potassium thiocarbonate bromate, ferrous ammonium sulphate, etc.) to stimulate the polymer chains, compared to irradiation grafting which employs radiation of high energy (γ-radiation) to obtain a free radical, followed by free radical polymerization [180].

An example of a water-soluble polymer that can be easily grafted is PEI, which exhibits high hydrophilicity, long flexible chains, high charge density [181], and abundant reactive groups (–NH_2_ and –NH), so that it can easily react with acid, acid chloride, epoxy, and isocyanate to modify the surface. In a recent study, C Zhang et al. successfully grafted L-cysteine (L-Cys), using PEI and CS as the carrier, obtaining two efficient, cheap, and readily available adsorbent materials chemically modified by the covalent bond of the backbone amino acids [182]. They had a good adsorption effect on Hg(II). The maximum Hg(II) adsorption capacities of PEI-Cys and CS-Cys of 231.2 mg/g and 407.4 mg/g were obtained, respectively. The results showed that L-cysteine methyl ester (an amino acid) can selectively adsorb Hg(II) in aqueous solution. The authors analyzed the adsorption behavior of Hg(II) on the two adsorbents, which can be well described by Langmuir isotherms, while the kinetics is pseudo-second order [182]. In another study, the group of NH Yusof et al. prepared CS-PEI balls by graft copolymerization under microwave irradiation and crosslinking with glutaraldehyde [183]. These were tested for the adsorption of Acid Red 27 dye from water. Crosslinking with glutaraldehyde greatly improved the mechanical and chemical properties of the obtained grafted and crosslinked CS granules (CS-PEI-GLA). Adsorption experiments were carried out in a batch system and optimized by varying the adsorbent dose, pH, initial dye concentration, and contact time. The kinetic model was described by the pseudo-second-order model, and the adsorption equilibrium was determined by the Langmuir isotherm model. The synthesized materials showed a maximum adsorption capacity of 48.3 mg/g at 27 °C. The dye adsorption mechanism was realized by electrostatic interactions [183]. For the removal of DS from water, new spherical polymeric adsorbents composed of crosslinked CS beads grafted with PEI were synthesized [184]. The crosslinking reaction improved the mechanical properties of the adsorbent, improving their handling. The presence of PEI polymer increased the number of functional groups and available adsorption sites on the surface of the adsorbent and improved the adsorption capacity for DS. The fabricated epichlorohydrin-PEI (EPCS@PEI) adsorbent demonstrated a maximum adsorption capacity of 253.32 mg/g and a removal efficiency of nearly 100% in the initial solution of 50 mg/L. The adsorption kinetics were described by the pseudo-second-order model, and the adsorption equilibrium was according to the Langmuir isotherm model. The material showed a good adsorption capacity even after recycling five times [184].

## 4. Different Composite Hydrogels Used for Water Purification

In order to highlight the importance of chitosan-based hydrogels, Table 4 shows some other different composite hydrogels based on natural biopolymers used for water purification.

Based on the data from Table 4 and our review, it can be emphasized that biopolymers, as “green” compounds from natural sources, can be used as a promising alternative for developing more sustainable materials and technologies for the removal of contaminants from wastewater and water purification.

## 5. CS-Based Hydrogels Used in Various Other Applications

**Scheme 2:**
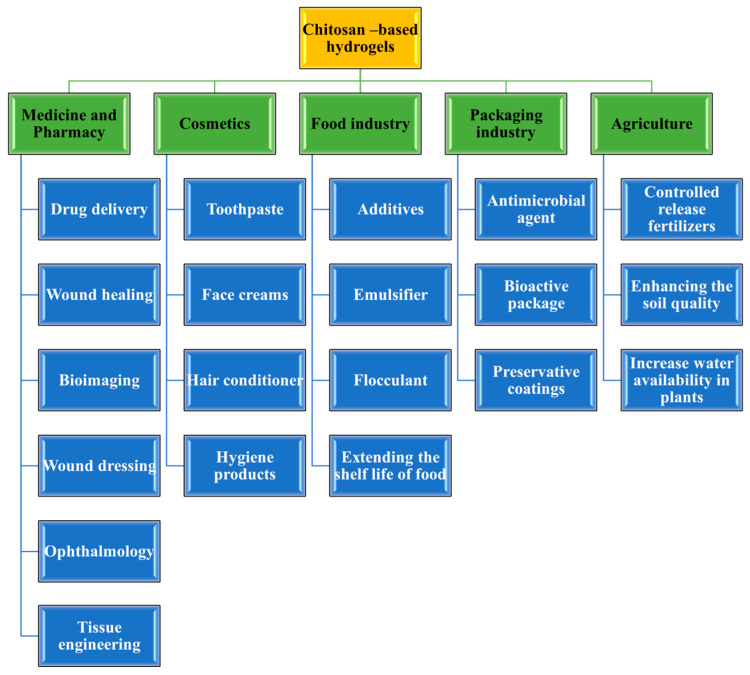
Schematic representation of chitosan-based hydrogels in various industries.

In recent period, CS-based hydrogels have attracted tremendous interest to be used in various applications other than industrial wastewater treatment. Their applications are based on the exceptional physical, mechanical, chemical, and antibacterial properties of CS, such as mucoadhesiveness, swelling ability, low immunogenicity, enzymatic biodegradability, similarity to host tissues, excellent biocompatibility and mechanical strength, pH-sensitivity, and antibacterial effect. CS-based hydrogels are used in several other fields, including medicine and pharmaceuticals, cosmetics, the packaging and food industries, and agriculture (Scheme 2).

Table 5 summarizes some examples of the recent use of CS-based hydrogels in various applications.

## 6. Conclusions and Prospective Studies

The versatility of CS has broad application prospects, and CS-based hydrogels can be designed and developed with several novel functions for various high-value applications in multiple fields. The use of biodegradable, innovative, and bio-derived resources to design efficient and inexpensive adsorbent materials has increased the potential for new perspectives in wastewater remediation. CS-based hydrogels have a high capacity against various types of water pollutants due to their biodegradability, inexpensiveness, abundance, adsorption performance, and reusability. The 3D network morphology of CS-based hydrogels makes them recyclable and suitable for different viable applications. The main advantages of these types of adsorbents for use in wastewater treatment are their outstanding adsorption capacity, easy reuse and recovery, together with better mechanical, thermal, and chemical stabilities.

The present review critically examined the numerous categories of CS-based hydrogel composites and their adsorption mechanism and efficiency in removing various compounds from wastewater. The extraordinary chemical and physical ability to be used in various ways of those compounds has stimulated scientists to advance new water decontamination technologies. Consequently, it has been suggested that future studies in this area should focus on emerging sustainable resources and encouraging the progress for strong, smart, biodegradable, reusable, and low-cost composites for possible further industrial, agricultural, and medicinal applications and for their commercialization.

## Figures and Tables

**Table 1:** Mechanisms of adsorption of chitosan-based hydrogels.

Adsorption Mechanism	Definition	Examples	Applications	References
hydrogen bonding	an intermolecular or intramolecular interaction with the formation of a pair of electrons–hydrogen bond acceptor	carboxymethyl cellulose-chitosan system (CMC-CS)	high removal efficiency of methylene blue (MB)	[76]
electrostatic interactions	the interaction between two oppositely charged molecules or/and atoms	carboxymethyl cellulose/chitosan hydrogel (CMC-CS)	removal of anionic and cationic dyes (MB and acid orange II)	[77]
ion exchanged	exchange of free mobile ions of an insoluble solid with different ions of a similar charge from the solution, the reaction being reversible	chitosan–gelatine ion exchanger with the addition of zirconium (IV)	an increased selectivity for cationic dyes—removal of MB	[78]
π–π interactions	noncovalent interactions by π-electron interactions between the acceptor and donor molecules	chitosan/carboxymethyl cellulose hydrogel using graphene oxide as chemical crosslinking agent	used to treat dyes from contaminated wastewater	[79]
coordination/chelation	the connection through a coordination bond of a metal ion with two or more coordination atoms (non-metal) in the same molecule to form a chelate ring structure	DNA-chitosan hydrogel	the removal of heavy metal ions, organic dyes, and pharmaceuticals	[80]
surface complexation	The interaction between an electron pair donor and electron acceptor in order to form various complexes	hydroxyapatite/chitosan composite (HAp-CS)	the removal of Congo red (CR)	[81]

**Table 2:** CS-based composite hydrogels for wastewater remediation.

Adsorbent	Pollutant	Adsorption Capacity (mg/g)/Adsorption Efficiencies (%)	Time/Equilibrium and Best-Fitted Model for Adsorption	References
CS magnetic-kaolin beads	Nitrate Phosphate	74.11/ 92.05	40 min Freundlich (pseudo-second-order kinetic model)	[115]
Imprinted magnetic CS beads	Cu(II)	78.1	180 min Langmuir (pseudo-second-order kinetic model)	[116]
Ion-imprinted crosslinked CS	Cd(II)	1.10 mmol/g	48 h Langmuir (pseudo-second-order kinetic model)	[117]
La-Fe incorporated CS beads	Cd(II) phosphate (calculated in P)	35.5/ 52.0	24 h 32 h Langmuir (pseudo-second-order kinetic model)	[118]
CS oligosaccharide	Cr(VI)	148.1	100 min Langmuir (pseudo-second-order kinetic model)	[119]
CS/orange peel	Cr(VI) Cu(II)	107.5 116.6	240 min Freundlich (pseudo-second-order kinetic model)	[120]
Crosslinked CS/sodium alginate/calcium ion	Pb(II) Cu(II) Cd(II)	176.50 70.83 81.25	8 h Elovich (pseudo-first-order kinetic model) (pseudo-second-order kinetic model)	[121]
Carbon dots crosslinked cellulose Nanofibril/CS	Cu(II) Cr(VI)	148.3 294.46	4 h Langmuir (pseudo-second-order kinetic model)	[122]
CS/CA/Bentonite	Pb(II) Cu(II) Cd(II)	434.89 115.30 102.38	1500 min Elovich (pseudo-second-order kinetic model)	[123]
CSGO (chitosan-graphene oxide)-R@IO (magnetically separable chitosanbased hydrogel system)	Cu(II)	119.5 at pH 6	180 min Langmuir (pseudo-second-order kinetic model)	[124]
Graphene oxide-poly(vinyl alcohol)- chitosan GO-PVA-CS	Cd(II) Ni(II)	172.11 70.37	16 h Langmuir (pseudo-second-order kinetic model)	[125]
Nitrogen-doped carbon dots-cellulose nanofibril-chitosan NCDs-CNF/CS	Cu(II) Cr(VI)	148.3 294.46	4 h Langmuir (pseudo-second-order kinetic model)	[126]
Millettia speciosa Champ cellulose-CS	Congo red (CR) Cu(II)	221.43 23.37	288 h, Freundlich (pseudo-second-order kinetic model) 24 h, Freundlich (pseudo-first-order kinetic model)	[127]
Graphene oxid-chitosan-Fe_3_O_4_ GO-CS-Fe_3_O_4_	Methylene blue (MB) Eriochrome Black T (EBT)	289 292	300 min Langmuir (pseudo-second-order kinetic model)	[128]
CS/aspartic acid CSAA-HG1 CSAA-HG2	Methylene blue (MB)	99.85% 99.88%	30–60 min Freundlich (pseudo-second-order kinetic model)	[129]
CS/montmorillonite	Methyl green (MG)	303.21	24 h Freundlich (pseudo-second-order kinetic model)	[130]
CS/oxalic acid	Reactive Red 195 RR195	110.7	16 h Redlich–Peterson	[131]
Polyacrylamide-chitosan-carbon PAM/CS/C PAMCS/MW-120	Acid Blue 113	151.7 255.5	60 min, Langmuir (pseudo-second-order kinetic model) Freundlich (pseudo-second-order kinetic model)	[132]
Biochar/CS	Ciprofloxacin Enrofloxacin	106.038 100.433	300 and 450 min Langmuir (pseudo-second-order kinetic model)	[133]
Pectin/O-carboxymethyl chitosan	Levofloxacin Delafloxacin	66.3–87.5% 45–53%	12 h SIPs model	[134]

**Table 3:** Advantages and disadvantages of different CS beads and their adsorption mechanisms.

Type of CS Beads	Advantages	Disadvantages	Type of Adsorbent/Pollutant	Mechanism	References
CS-based composite beads	easy to prepare, no mass loss, high surface area, and good reusability	hard selection of low-cost materials	CS/Fe-hydroxyapatite beads	chemisorption	[136]
			CS/Ag-hydroxyapatite nanocomposite beads	ion exchange	[137]
			CS/waste active sludge char beads	chelation	[138]
			La-Fe incorporated CS beads	electrostatic interaction and ligand exchange	[139]
			CS/lanthanum hydrogel beads	electrostatic attraction and ion exchange	[140]
Functionalized CS beads	high surface area, a multitude of available active sites, and good reusability	unclear adsorption mechanism	ethylenediamine tetraacetic acid grafted/polyvinyl alcohol/CS beads	chemisorption	[141]
			functional β-cyclodextrin CS beads	electrostatic interaction	[142]
			triethylenetetramine-CS/alginate composite beads	electrostatic interaction, hydrogen bonding, redox reaction, chelation, and cation–anion exchange	[143]
			novel aliquot-336 impregnated CS conjugated beads	electrostatic interaction	[144]
			CS beads impregnated hexadecyl amine	chemisorption	[145]
Metal–organic framework-based CS beads	higher porosity and surface area	low stability and low mechanical strength	MIL-101(Fe)-CS-cyclodextrin nanosponge	-	[146]
			copper-benzenetricarboxylate (Cu-BTC), polyacrylonitrile (PAN), and CS	physical adsorption	[147]
			Al-MOF@CS composites with the MOFs Alfum and MIL-160	-	[148]
			CS/PEO@MOF-5 membrane	-	[149]
			PAN/CS	complexation	[150]
Magnetic CS beads	good mechanical strength, good stability, good recovery, and higher surface area	lower reusability	magnetic kaolinite immobilized CS beads	-	[151]
			magnetic kaolin-embedded CS beads	physical adsorption	[152]
			magnetic Fe_3_O_4_/graphene oxide/CS beads	-	[153]
			magnetic cationic polymer CS beads	electrostatic interaction and ion exchange	[154]
Co-polymeric CS beads	a good adsorption capacity	lower stability	PEI-grafted CS beads	-	[155]
			graft copolymerized CS with acetylacetone	complexation	[156]
			epichlorohydrin-modified CS-2,4-dichlorobenzaldehyde composite	homogeneous monolayer chemical adsorption	[157]
			Zr^4+^ and glutaraldehyde crosslinked PEI functionalized CS	electrostatic action, complexation, and reduction	[158]
Imprinted CS beads	good reusability and greater efficiency	unclear adsorption mechanism	ion-imprinted crosslinked CS	chemisorption	[117]
			imprinted CS-acrylamide	hydrogen bond and electrostatic attraction	[156]
			imprinted magnetic CS beads	enthalpy controlled process	[116]
			ion-imprinted tetraethylenepentamine modified CS beads	diffusion process	[159]

**Table 4:** Composite hydrogels for wastewater remediation.

Adsorbent	Pollutant	Adsorption Capacity (mg/g)	References
Xanthan gum-poly(acrylic acid-cloisite15A semi-IPN hydrogel (XG/PAA/Cloisite15A)	Co(II) Cu(II) Ni(II)	436.62 530.14 511.74	[185]
Poly(acrylamide) PAA-g-starch/PAA semi-IPN cryogels	Methylene blue (MB)	667.7	[186]
alginate/polyethyleneimine (ALG/PEI) hydrogels	Cu(II) Pb(II)	322.6 344.8	[187]
polyaniline/sodium alginate hydrogel (PANI/ALG)	MB Rhodamine B Orange-II Methyl orange	555.5 434.78 476.19 146.66	[188]
Xanthan gum-2-acrylamido-2-methly propane sulfonic acid-acrylamide XG-g-P(AMPS-co-AAm)	MB	384.62	[189]
hydroxyethyl starch/p(3-(acrylamidopropyl) trimethyl ammonium chloride (HESt/P(APTMACl)) hydrogel HESt/PAA hydrogels hydroxyethyl starch/p(acrylamide) (HESt/PAAm) hydrogels	Methyl violet Methyl orange Methyl violet Methyl orange Methyl violet Methyl orange	6.62 238.1 185.2 2.84 9.17 3.33	[190]
Polydopamine-montmorillonite-pullulan PDA/MMT/PUL hydrogel composites	Crystal violet	112.45	[191]
hydrogel nanocomposites based on methacrylated carboxymethyl cellulose (M-CMC) and lignosulfonate-derived carbonaceous products (All-lignocellulose)	MB Cu(II)	350 145	[192]
chondroitin sulfate/MCM-41 (chondroitin sulfate-mesoporous silica) hybrid hydrogels	MB	3982 ± 123.6	[193]

**Table 5:** Examples of CS-based hydrogel applications.

Domains	Applications	Examples	References
medicine and pharmaceutics	treating skin wounds and accelerated wound healing	- injectable hydrogel for dressing wound healing (hydrophobically modified chitosan/oxidized-dextran hydrogel) - sponge dressing as rapid and efficient hemostatic agents (carbon nanotube/hydroxybutyl chitosan) - dressings for infected wound treatment	[194,195,196,197,198]
	wound dressing	- biosynthesized zinc oxide nanoparticles incorporated into a chitosan hydrogel matrix functionalized with propolis extract	[197,199]
	drug delivery	- temperature-dependent thermoreversible supramolecular hydrogel for applications in the field of gastrointestinal drug release (photo-crosslinkable glycol chitosan thermogel, graphene oxide reinforced chitosan hydrogel)	[197,200,201,202,203]
	lens system for eyes	release of various ophthalmic drugs (4-arm polyethylene glycol with aldehyde end groups (4-arm PEG-CHO) with glycol chitosan (GC))	[197,204]
	bones and tissues engineering	- injectable delivery system and 3D cell culture to fill the irregular shaped gaps and defects of internal tissues without any surgery (chitosan/laponite hydrogel) - sequential delivery of the anti-inflammatory drug aspirin and osteogenic bone morphogenetic protein 2 (BMP-2)	[205,206]
	cartilage repair	hydrogels for in vivo cartilage regeneration (chitosan-hyaluronic acid hydrogel)	[207,208,209,210]
	neural tissue	injectable thermoresponsive hydrogels for neural tissue engineering (chitosan/β-glycerophosphate hydrogels)	[211,212]
	engineering	3D materials for tissue engineering applications	[213]
cosmetics	skin care products	- base for cosmetic masks - deodorizing agent - preventive water loss agent - antibacterial and antioxidant activity—hydrating agent	[214,215]
	hair care products	- film forming - hair fixing	
	hygiene	- superabsorbent materials - female hygiene products (hydrogel nanocomposites of silver nanoparticles (AgNPs)/chitosan/polyvinyl alcohol/polyethylene glycol)	
food industries	food preservatives	- antifungal coatings - natural food preservatives - as impregnating chitosan solution for purpose preserving and storing food by spreading or spraying	[216,217,218,219,220,221,222,223,224,225,226]
	dietary ingredient	- additive in food and beverages - flavor enhancer in food (chitosan and *Mentha piperita* L. essential oil)	
	color stabilization in foods	- encapsulant for flavor encapsulation - maintain the desired structure and texture	
	controlling and stabilizing agent	- antibacterial agent - stabilizer for food texture	
agriculture	removal of pesticides and herbicides	antifungal control	[217,227,228,229,230,231,232,233]
	plant growth	- as nanofertilizers (Chitosan-Montmorillonite (MMT) Nano-Composite Hydrogel) - release plant growth regulators, and vitamins	
	enhancing the soil quality	for biologically transmitting active nutrients	
packaging industry	packaging for food	- food packaging (Incorporating Purple-Fleshed Sweet Potato Extract into Chitosan Matrix)	[234,235,236,237,238]
	active/preservative coatings	- biofilms coatings for the protection of fruits - biosensors	

## Data Availability

Not applicable.
